# Evidence for abnormal cytokine expression in Gulf War Illness: A preliminary analysis of daily immune monitoring data

**DOI:** 10.1186/s12865-015-0122-z

**Published:** 2015-09-30

**Authors:** Luke Parkitny, Stephanie Middleton, Katharine Baker, Jarred Younger

**Affiliations:** Neuroinflammation, Pain & Fatigue Lab, University of Alabama at Birmingham, Birmingham, AL USA; Department of Anesthesia, Perioperative, and Pain Medicine, Stanford University, Palo Alto, CA USA; Experimental Neuropsychology Research Unit, School of Psychological Sciences, Monash University, Melbourne, Australia

**Keywords:** Gulf War, Fatigue, Inflammation, Cytokine, Daily, Longitudinal, Immune

## Abstract

**Background:**

Gulf War Illness (GWI) is a clinically heterogeneous chronic condition that affects many veterans of the 1990–1991 Persian Gulf War. One of the most prevalent and debilitating symptoms of GWI is abnormal fatigue. The mechanisms underlying GWI generally, and fatigue symptoms specifically, have yet to be conclusively identified, although immune system abnormalities are suspected to be involved. The first goal of this immune monitoring study was to determine if GWI is associated with higher absolute levels and daily variability of pro-inflammatory immune factors. The second goal was to explore the relationship between day-to-day immune marker fluctuations and daily self-reported fatigue severity.

**Methods:**

We recruited veterans with GWI and healthy veteran control (HV) participants to provide self-reported fatigue severity data and blood samples, over 25 consecutive days. We profiled inflammatory processes by using a longitudinal, daily immune-monitoring approach. For each day, serum cytokine and chemokine concentrations were determined using multiplex assays.

**Results:**

Seven veterans with GWI and eight healthy veteran control (HV) participants completed the study protocol. We found that GWI was associated with higher variability in the expression of eotaxin-1 (p < 0.001). For GWI participants, higher fatigue severity days were associated with greater IL-1β (p = 0.008) and IL-15 (p < 0.001).

**Conclusions:**

Our findings provide preliminary evidence that the immune system is involved in the pathophysiology of GWI. Longitudinal immune profiling approaches may be helpful in discovering targets for novel therapies in conditions such as GWI.

## Background

Gulf War Illness (GWI) is a chronic condition that affects military veterans of the 1990 – 1991 Persian Gulf War. The clinical manifestation of GWI is heterogeneous; symptoms can include fatigue, cognitive problems, widespread pain, gastrointestinal issues, respiratory difficulties, and dermatological complaints [1, 2].

The mechanisms underlying GWI have not been properly resolved, although abnormal immune function has been proposed to play a role. One etiological hypothesis suggests that immune challenges due to various environmental exposures, possibly exacerbated by conditions of battle stress, may have resulted in chronic dysregulation of the immune system [3–7]. Unfortunately, while a specific immunological signature for GWI holds great diagnostic appeal, the question whether GWI is associated with immune dysregulation has not been conclusively answered. Some groups have suggested that the underlying immune dysregulation involves a polarization towards either the T-helper 2 (Th2) [8] or Th1 [9, 10] direction. It has also been suggested that interleukin (IL)-1β is involved [11] as it is an important driver of sickness responses [12] that can resemble the symptoms of GWI. However this is challenged by at least one study, which reported that concentrations of IL-1β in plasma and following *ex vivo* stimulation of peripheral blood mononuclear cells were no different in GWI than in a veteran control group [13]. Results from whole blood analyses have suggested normal lymphocyte function in GWI [14], while others have reported that GWI may be associated with changes in the populations and function of B and T immune cells [15–19]. Routine clinical and rheumatological blood tests have generally been shown to be normal [18, 20], including tests for autoimmune conditions, such as the antinuclear antibody (ANA) [21].

Although activated immune processes may underlie the symptoms of GWI, research results to date remain inconclusive. One possible issue with present data is that, with a few notable exceptions [15–19], most past attempts at exploring immune pathology in GWI have involved cross-sectional, patient-versus-control designs. Such approaches may be insensitive to pathophysiological inflammation processes if significant day-to-day fluctuations are common in the patient group. Factors that drive disease processes may also be missed if those factors operate in clinically “normal” ranges, but still drive symptoms because of sensitized downstream targets. For those reasons, experimental exercise-based stimulation paradigms have been employed to assess immune system function [16]. We have also proposed a daily immune monitoring approach, which allows us to examine immune responses to typical daily stimuli and experiences [22]. Daily sampling of immune variables also allows more accurate individual means to be computed, variability across time to be calculated, and associations between analyte fluctuations and symptom severity to be explored. Longitudinal immune monitoring designs therefore allow the immune system to be examined in ways not possible with cross-sectional designs.

The overall goal of this preliminary analysis was to test whether GWI is associated with either abnormal concentrations or abnormal fluctuations in pro-inflammatory and anti-inflammatory immune markers. In this longitudinal study, we examined blood sera collected over 25 consecutive days from veterans who met the Kansas GWI case definition criteria [23] and from healthy veterans of the Gulf War (HV). Three hypotheses were generated for the analyses. First, that serum pro-inflammatory cytokines would be higher in GWI than in HV. Second, that cytokine fluctuations would be greater in GWI than in HV. Third, that daily self-reported fatigue in GWI individuals would covary with the concentrations of pro-inflammatory cytokines.s. We focused on fatigue because it has been identified as one of the most significant and common problems in GWI [23–26]. Although involving few participants, this study utilized intensive longitudinal data to explore unique questions about the role of systemic inflammation in the symptoms of GWI.

## Results

### Participants

We included seven GWI and eight HV participants in this study. Five additional GWI participants were recruited but subsequently excluded from the study: three due to exclusionary blood test results, including a positive and high ANA, a high thyroid-stimulating hormone (TSH) level indicating a thyroid disorder, and a high erythrocyte sedimentation rate (ESR) and C-reactive protein (CRP) level indicating a possible autoimmune disorder; one due to a HADS depression score above the study cut-off; and one because he did not experience any symptoms during the study period, which was highly atypical of his usual symptomatology. Of the 15 included participants, one GWI participant withdrew after 19 consecutive blood draws, however we resolved that he had contributed enough data to be included in the analyses. All other participants had blood draws collected as described in the protocol. The participant demographics and relevant baseline measures are shown in Table 1.

**Table 1:** Participant demographic information and baseline symptom and medical profile

Participant	Group	Age (yrs)	Ethnicity	Alcohol intake/week (standard drinks)	Liver disease	Average pain (0–10 NRS)	HADS Anxiety score	HADS Depression score	Fatigue mean ± .d.	WBC *K/μL*	Platelets *K/μL*	CRP *mg/dL*	ESR *mm/hr*	ANAS	TSH mIU/L	RHF
1	HV	47	Caucasian	9	neg.	0	1	1	0 ± 0	6.3	242	<0.2	0	neg.	1.11	neg.
2	HV	50	Asian	1	neg.	0	0	1	0 ± 0	4.7	219	<0.2	1	neg.	1.24	neg.
3	HV	42	Caucasian	15	neg.	1	1	3	17 ± 6	5.1	218	<0.2	0	neg.	0.86	neg.
4	HV	41	Caucasian	6	neg.	0	6	1	0 ± 0	7.3	212	<0.2	4	neg.	1.04	neg.
5	HV	57	Caucasian	0	neg.	0	2	0	2 ± 3	6.3	239	0.8	30	neg.	0.96	neg.
6	HV	54	Caucasian	1	neg.	3	14	11	65 ± 25	7.0	234	<0.2	5	neg.	1.83	neg.
7	HV	44	Caucasian	1	neg.	2	5	4	0 ± 0	9.4	189	0.5	5	neg.	2.42	neg.
8	HV	48	Latino	0	neg.	0	3	1	5 ± 16	7.9	332	0.6	2	neg.	0.96	neg.
9	GWI	41	Caucasian	42^*^	neg.	6	8	7	50 ± 14	10.1	262	0.2	1	1:80	1.27	neg.
10	GWI	42	Caucasian	1	neg.	2	11	10	25 ± 15	6.6	212	<0.2	2	neg.	1.98	neg.
11	GWI	44	Pacific Islander	1^*^	neg.	2	6	7	65 ± 12	12.3	284	1.0	1	neg.	2.22	neg.
12	GWI	48	Caucasian	0	neg.	5	8	3	37 ± 15	7.4	256	<0.2	2	neg.	1.49	neg.
13	GWI	48	Asian	0	neg.	2	11	6	11 ± 11	4.4	188	<0.2	9	neg.	1.90	neg.
14	GWI	47	Caucasian	7	neg.	2	5	1	21 ± 16	5.8	191	<0.2	2	neg.	1.08	neg.
15	GWI	43	Caucasian	6	neg.	3	9	4	24 ± 19	5.3	278	<0.2	2	neg.	0.63	neg.

Except for fatigue, all data shown were obtained during each participant’s screening session. Fatigue means and standard deviations were calculated for each participant from the self-report scores provided during the immune-monitoring phase. HADS = Hospital Anxiety and Depression Score. Blood test results include white blood cell count (WBC), platelets, C-reactive protein (CRP), erythrocyte sedimentation rate (ESR), antinuclear antibody test (ANA), thyroid stimulating hormone (TSH), and rheumatoid factor test (RHF). Neg. = negative. ^*^Participant 9 currently diagnosed with alcohol addiction; participant 11 recovered from alcohol addiction. There was no significant difference in alcohol intake between the healthy (median = 1; IQR = 6) and the GWI (median = 1; IQR = 7) groups (U = 27.0, *p =* 0.955). There was a significant difference in HADS-Anxiety (t = −2.254, *p =* 0.042) between HV (mean = 4.0, sd = 4.5) and GWI (mean = 8.3, sd = 2.3), but not for depression (t = −1.559, *p =* 0.143)

### Main results

First, the between-group (GWI versus HV) differences in serum cytokine concentrations were assessed (Table 2; column 7). We found no significant main differences in the levels of serum-expressed cytokines in the GWI and HV groups.

**Table 2:** Results of statistical tests comparing cytokine expression in the GWI and HV groups

Immune biomarker	GWI		HV		CV: GWI *vs.* HV t-test results	GWI *vs.* HV GEE results	GWI fatigue-cytokine LMM results
	Conc. mean (sd)	CV mean (sd)	Conc. mean (sd)	CV mean (sd)			
BDNF	23.36 (8.84)	29.78 (22.28)	23.97 (6.79)	16.86 (6.24)	t(13) = −1.578, *p* = 0.138	*χ* ^2^ = 0.068, *p* = 0.794	F = 0.911, *p* = 0.341
Eotaxin-1	168.94 (66.21)	23.89 (3.99)	227.17 (129.9)	15.27 (1.78)	t(11) = −5.173, *p* ≤ 0.001	*χ* ^2^ = 3.221, *p* = 0.073	F = 3.467, *p* = 0.065
Factor VII	391.89 (124.38)	9.71 (4.75)	407.54 (54.29)	7.74 (1.63)	t(13) = −1.104, *p* = 0.290	*χ* ^2^ = 0.180, *p* = 0.671	F = 0.849, *p* = 0.358
ICAM-1	118.89 (32.35)	10.6 (3.58)	125.21 (34.4)	9.59 (2.52)	t(13) = −0.639, *p* = 0.534	*χ* ^2^ = 0.429, *p* = 0.512	F = 0.035, *p* = 0.852
IL-1β	4.49 (0.7)	15.61 (3.24)	3.84 (0.39)	10.49 (3.09)	t(12) = −3.011, *p* = 0.011	*χ* ^2^ = 5.204, *p* = 0.023	F = 7.238, *p * = 0.008
IL-1Ra	579.28 (184.39)	25.68 (7.62)	636.16 (106.9)	21.87 (3.75)	t(13) = −1.257, *p* = 0.231	*χ* ^2^ = 0.161, *p* = 0.688	F = 2.140, *p* = 0.145
IL-8	7.06 (1.42)	27.42 (15.1)	7.54 (3.88)	28.21 (13.9)	t(13) = 0.106, *p* = 0.917	*χ* ^2^ = 0.483, *p* = 0.487	F = 5.046, p = 0.026
IL-10	6.09 (0.57)	17.23 (8.15)	5.69 (0.26)	16.07 (7.39)	t(8) = −0.235, *p* = 0.820	*χ* ^2^ = 2.890, *p* = 0.089	F = 0.837, *p* = 0.364
IL-12p40	0.39 (0.1)	19.03 (2.34)	0.46 (0.07)	15.98 (3.1)	t(13) = −2.126, *p* = 0.053	*χ* ^2^ = 1.845, *p* = 0.174	F = 3.418, *p* = 0.066
IL-15	0.76 (0.1)	15.6 (2.77)	0.73 (0.1)	14.49 (3.5)	t(11) = −0.600, *p* = 0.561	*χ* ^2^ = 1.366, *p* = 0.242	F = 21.951, *p* ≤ 0.001
IL-17	4.02 (0.27)	21.34 (12.13)	3.78 (0.19)	5.51 (9.55)	t(4) = −1.776, *p* = 0.150	*χ* ^2^ = 2.379, *p* = 0.123	F = 0.391, *p* = 0.536
IL-18	287.44 (83.16)	12.79 (3.72)	243.66 (102.56)	10.38 (3.8)	t(13) = −1.236, *p* = 0.238	*χ* ^2^ = 0.670, *p* = 0.413	F = 0.973, *p* = 0.325
IL-23	1.93 (0.11)	13.58 (3.76)	1.9 (0.13)	9.29 (2.84)	t(10) = −2.259, *p* = 0.047	*χ* ^2^ = 1.280, *p* = 0.258	F = 1.320, *p* = 0.255
MIP-1α	35.24 (2.24)	7.26 (3.96)	38.87 (6.28)	7.94 (4.18)	t(6) = 0.237, *p* = 0.821	*χ* ^2^ = 0.243, *p* = 0.622	F = 0.367, *p* = 0.547
MIP-1β	246.95 (82.29)	21.41 (7.05)	342.66 (125.03)	13.58 (4.42)	t(13) = −2.536, *p* = 0.030	*χ* ^2^ = 3.121, *p* = 0.077	F = 4.451, *p* = 0.036
MMP-3	12.32 (5.2)	18.25 (7.12)	14.91 (5.81)	17.19 (8.39)	t(13) = −0.262, *p* = 0.797	*χ* ^2^ = 0.745, *p* = 0.388	F = 1.140, *p* = 0.287
MMP-9	58.31 (15.98)	21.9 (8)	65.44 (28.67)	18.87 (3.42)	t(13) = −0.977, *p* = 0.346	*χ* ^2^ = 0.428, *p* = 0.513	F = 0.865, *p* = 0.354
MCP-1	295.95 (173.89)	21.75 (7.38)	419.04 (167.77)	18.07 (8.38)	t(13) = −0.897, *p* = 0.386	*χ* ^2^ = 2.704, *p* = 0.100	F = 1.142, *p* = 0.287
SCF	400.74 (98.01)	16.05 (3.18)	496.78 (80.29)	13.31 (2.31)	t(13) = −1.925, *p* = 0.076	*χ* ^2^ = 4.302, *p* = 0.038	F = 2.560, *p* = 0.111
VEGF	161.42 (82.29)	19.92 (9.36)	190.26 (86.36)	13.64 (4.62)	t(13) = −1.684, *p* = 0.116	*χ* ^2^ = 1.079, *p* = 0.299	F = 4.370, *p* = 0.038
Leptin	13.51 (6.25)	18.09 (4.4)	12.59 (10.22)	15.68 (3.47)	t(13) = −1.182, *p* = 0.258	*χ* ^2^ = 0.003, *p* = 0.958	F = 0.405, *p* = 0.525

For each analyte: columns 2–5 show the mean and standard deviation (sd) of the cytokine concentrations (conc.) and the day-to-day cytokine fluctuations (presented as the coefficient of variation or CV); column 6 shows the results of group comparison tests of the CVs between GWI and HV; column 7 shows the results of group comparisons of serum concentrations between GWI and HV; the last column shows the results of tests for associations between cytokine and symptom variability in GWI. Statistically significant results are bolded and underlined (at *p*=0.0098 which is α=0.05 adjusted for the expected false discovery rate in 93 statistical tests).

Second, we contrasted the magnitude of day-to-day fluctuations in serum cytokine concentrations between the GWI and HV groups (Table 2; column 6). Compared to HV, GWI participants had higher variability in the expression of eotaxin-1 (coefficient of variation (CV) = 23.89 ± 3.99 % *vs.* 15.27 ± 1.78 %; *p* < 0.001). No other analytes were more variable in the HV group than in the GWI group.

Finally, in the GWI group only, we tested for associations between analytes and daily fatigue severity (Table 2; column 8). Fatigue severity was positively associated with IL-1β (F = 7.238, *p =* 0.008) and IL-15 (F = 21.951, *p* < 0.001).

**Fig. 1:**
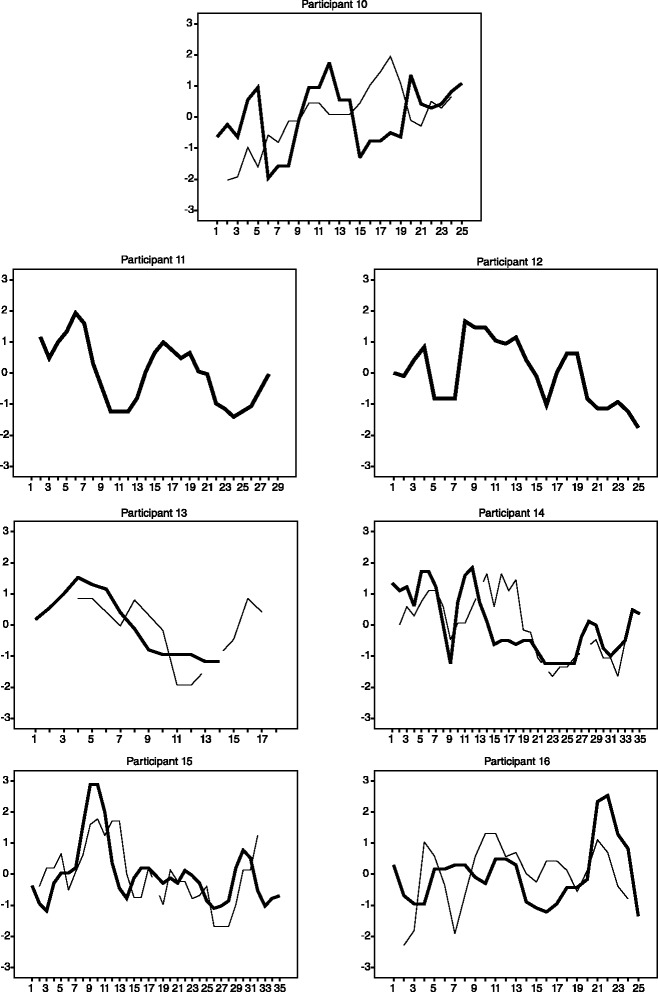
z-scored 3-day-smoothed serum IL-15 concentration (thin line) plotted against z-scored 3-day-smoothed daily self-reported fatigue (thick line), by participant. IL-15 concentration/fatigue severity are represented on the y-axis. Time is represented on the x-axis. IL-15 was selected for display as our statistical tests suggested that it was most significantly associated with fatigue severity fluctuations. Two participants did not express IL-15 at concentrations that were measurable

In all analyses, significance was determined using a false discovery rate (FDR) corrected threshold of *p =* 0.0098. Results of all analyses are shown in Table 2. Because in the GWI cohort, the levels of IL-15 were most strongly correlated with day-to-day changes in fatigue severity, we have also presented time-series plots that show the relationship between IL-15 and fatigue for each individual participant (Fig. 1).

## Discussion

The goal of this study was to explore inflammatory dysregulation as a potential component of the pathophysiology of GWI, by using a daily immune monitoring approach. We explored differences in the absolute concentrations and fluctuations of cytokines in GWI individuals and healthy Gulf War veteran controls. We also explored the relationship between daily cytokine fluctuations and changes in fatigue severity in individuals with GWI. We found that, compared to HV, GWI was associated with higher variability of eotaxin-1. For GWI participants, higher fatigue days were associated with greater concentrations of IL-1β and IL-15. As shown in Fig. 1, we observed that the relationships between IL-15 and fatigue over time vary considerably across individuals, however overall moderate positive relationships between the two variables can be seen. Combined, our results appear to support the hypothesis that the pathophysiology of GWI involves immune dysregulation.

### Main study findings

The cytokine IL-1β is a classic pro-inflammatory cytokine that is inhibited by the release of its receptor antagonist IL-1Ra [27]. That IL-1Ra expression was not found to be different in GWI or to be associated with fatigue suggests that a pro-inflammatory increased IL-1β/IL-1Ra ratio may be associated with fatigue. IL-1β is an important driver of the classic sickness response, which includes profound fatigue in both animals and humans [28]. Previous reports have been published of higher circulating levels of IL-1 in patients with myalgic encephalomyelitis/chronic fatigue syndrome [29–31] and that, in some medical conditions, the inhibition of IL-1 is associated with decreased fatigue [32, 33]. While we did not find increased concentrations of IL-1β in blood obtained from people with GWI, in agreement with prior work in this condition [13], we found that the levels of this cytokine were associated with fatigue. This offers some support to an early theory which suggested that IL-1β may play an important role in the pathophysiology of GWI [11].

IL-15 is a more recently discovered pro-inflammatory cytokine with angiogenic [34], anabolic muscle [35], T-cell activating [36], and microglial activating [37] effects. Prior work has shown that chronic fatigue syndrome may be associated with reduced IL-15 and, in turn, reduced natural killer cell activity [38]. A reduced IL-15 response to high-intensity exercise, but not at rest, has also been shown in males with GWI [39]. In the present study, we did not find any differences in the expression of IL-15 between GWI and HV, but its concentration was strongly and positively associated with fatigue. The mechanism of this is not clear. However, based on prior research that reported changes in CD8+ T-cell expression and function in GWI and chronic fatigue syndrome [19, 40], the changes in IL-15 expression may suggest that daily fatigue is associated with an altered immune response to activity and other stressors. Because we did not test immune cell populations, future work should also consider testing T-cell populations in addition to circulating cytokines to determine the mechanism of action.

Finally, we found that GWI individuals also showed abnormally high fluctuations in eotaxin-1 (CCL11). Eotaxin-1 is a chemotactic cytokine whose role in allergy has been well-defined. More recently, it has been suggested that eotaxin-1 may impair cognitive function through an inhibitory effect on neurogenesis [41]. However the role that this chemokine may play in GWI is not clear.

While this study does not elucidate the specific mechanisms through which these cytokines and fatigue are related, our data suggest that this may take place through a pro-inflammatory modulation of immune function in response to day-to-day activity. Since we did not find increased concentrations of IL-1β and IL-15 in GWI, our data also suggest that GWI is associated with a more labile immune system and perhaps a change in sensitivity to the expression of these cytokines. One possibility underlying these findings is that the immune system of people with GWI may be more reactive to daily stressors. This appears consistent with the hyper-reactive immune responses to exercise in people with GWI that were identified in prior work [15, 17, 19].

Interestingly, the present results contrast our earlier finding that the adipokine leptin is associated with self-reported fatigue in many people with chronic fatigue syndrome/myalgic encephalomyelitis [22]. First, it is possible that while fatigue is a distinct symptom, its underlying mechanisms may differ between people and among the various medical conditions. Second, the relationships between the immune modulators and fatigue may be more complex than those tested in the present study. We will aim to explore these possibilities in future studies.

### Study strengths and limitations

We highlight four limitations of this study. First, the participant sample size was small. The potential loss of power due to a small sample size was mitigated by the intensive longitudinal format of the study, yielding significant power through within-person repeated measures. The daily immune monitoring approach provides a rich dataset that allows unique questions to be asked of the immune system, including the within-person relationships between clinical and physiological variables. However we also suggest caution due to the unknown generalizability of our findings; future validation in an independent sample will help in resolving this uncertainty.

The second potential limitation of this study is related to the selection of fatigue as a dependent variable. Although GWI is a heterogeneous condition, due to the small sample size we avoided the testing of complex disease models and only focused our attention on fatigue because of its importance in the condition. However, it is possible that other relationships exist between inflammatory processes and other symptoms of GWI such as pain or cognitive problems.

Our third concern lies in the finding that the GWI group reported significantly more anxiety at baseline than did the HV group. It is possible that anxiety may play a role in GWI and that it may be a confounder of the presented relationships. We will investigate this possibility in our future research with a larger cohort.

Finally, while we collected information about specific exposure variables such as vaccination and environmental toxins encountered during deployment, we did not control for these variables in the analyses. We made this decision because accurate records of these exposures are not available. However, we attempted to control for this methodologically by using a control group that was composed of Gulf War veterans who did not experience GWI.

### Methodological considerations and recommendations

In this study, we used the relatively rare approach of collecting blood samples daily in GWI individuals and controls. While more demanding of time and resources, the longitudinal approach has two main advantages. First, serial longitudinal measurements are better than a single cross-sectional snapshot at capturing the functional immune profile of a participant. Second, because longitudinal studies obtain a series of observations that are taken over a representative period, they allow for the testing of causal relationships. Rheumatological and non-rheumatological inflammatory conditions are associated with significant low and high frequency variability in symptoms such as fatigue [42–45]. Frequent longitudinal assessments can allow those fluctuations to be described, and allow more complex relationships (such as time-lagged) to be explored.

## Conclusions

A limited understanding of the pathophysiology of GWI has impeded the development of specific and effective therapeutics. Importantly, the precise relationship between immune function and GWI symptoms is not known. In this study, we reported a potential relationship between GWI and serum-concentrations of eotaxin-1. We also found a temporal relationship between serum-concentrations of IL-1β and IL-15 and symptoms of fatigue in GWI.

## Methods

### Participant recruitment and consent

Study procedures were conducted as approved by the Institutional Review Board at Stanford University. All participants provided written informed consent including consent for publication of data. Participants were recruited through radio advertisements broadcast in the San Francisco Bay Area, advertisements on Craigslist, online support groups, and advertisements at the Veterans Affairs Palo Alto Health Care System.

Participant inclusion and exclusion criteria were initially determined following a phone pre-screening interview. Males between the ages of 39 and 65 were considered for this study. Potential participants were excluded for current use of opioid medications, significant psychological comorbidities, current involvement in litigation or worker’s compensation claims, current use of blood thinning medications, or current regular use of any anti-inflammatory medication (such as aspirin, ibuprofen, or naproxen) which may have confounded the inflammatory data. Participants were also required to provide 25 consecutive daily blood draws, either at Stanford University or through a mobile phlebotomy service (Health Exams Inc., Burlingame, CA).

Secondary screening was conducted in person at the Stanford Adult and Pediatric Pain Laboratory. During this appointment, detailed participant demographic and medical history information were collected. Individuals were admitted into one of two study groups, GWI or HV. Participants included in the GWI group met the Kansas Gulf War Illness case definition criteria [23]. In brief, these criteria restrict the diagnosis to veterans who were deployed to the Persian Gulf and subsequently reported moderate to severe symptoms in three out of six symptom categories: fatigue or sleep problems; pain symptoms; neurologic, cognitive or mood symptoms; gastrointestinal symptoms; respiratory symptoms; and skin symptoms [23]. Participants in the HV group were required to have been deployed during the Gulf War, to be free of any current major medical diagnoses, and to not report daily pain or fatigue at the time of the assessment. Exclusionary criteria for all participants included a depression subscale score of ≥16 on the Hospital Anxiety and Depression Scale (HADS) [46], a score of ≥50 on the Military Post Traumatic Stress Score [47], or current use of opioid analgesics or anti-inflammatories. Individuals were also excluded if screening blood tests showed abnormal values of thyroid hormone, an ANA ratio >1:80, erythrocyte sedimentation rate (ESR) >60 mm/h, positive rheumatoid factor, C-reactive protein over 1.0 mg/L, or clinically abnormal results of a complete blood count test according to the testing laboratory’s reference ranges.

### Study design

We used an observational longitudinal design to test the role of immune function in GWI. The 42-day study began with the screening session during which participants were provided with the data collection device and instructed in its use. The study consisted of a two-week baseline symptom reporting phase, followed by 25 consecutive days of symptom reporting and venous blood draws, and ended with a three day follow-up phase during which participants reported their daily symptoms. Participants could miss up to 2 days consecutively under special circumstances, in which case additional days were added to the end so that 25 days total were assessed.

### Questionnaire data collection

Baseline questionnaires used for screening purposes included the Kansas Gulf War Veterans Health Project Questionnaire [23] and the Hospital Anxiety and Depression Scale [46]. Daily GWI symptoms were scored by the participants on 0–100 visual analog scales (VAS), using commercial survey software (Dooblo SurveyToGo, Kefar Sava, Israel) on an android-based device. Fatigue, the primary dependent variable, was assessed by asking, “How fatigued have you felt today?” The VAS was anchored on the left by “No fatigue at all” and on the right by “Severe fatigue”. Similar 101-point VAS were used to collect information about other symptoms in the GWI diagnostic criteria, but were not analyzed in this study. The daily self-report measures were completed by the participants in the afternoon or evening.

### Immune data collection

During the 25-day immune monitoring phase, blood was collected by trained phlebotomists or research nurses at the Stanford Clinical and Translational Research Unit (CTRU) or a mobile phlebotomy service (Health Exams Inc). Venous blood was generally collected from the cubital fossa using a 23-gauge butterfly needle into two 4 cc serum separating tubes. The venipuncture site was rotated daily to minimize participant discomfort and to maintain local vein integrity. Obtained blood samples were coagulated at room temperature for 30 min, centrifuged at 350 g for 15 min, and the serum layer was then aliquotted into vials for storage at −80 °C. Phlebotomy visits were scheduled within a two-hour window for each participant, to minimize the effect of diurnal variation on cytokine concentrations [48, 49]. Blood pressure, heart rate, and body temperature were also collected during each visit to monitor for signs of emerging acute illness.

Concentrations of cytokines and chemokines in serum samples were determined by Myriad RBM, Inc. using a standardized protocol. In short, Tecan EVO® robots were used to combine an aliquot of each sample with the capture microspheres and incubated for one hour at room temperature. Multiplexed cocktails of biotinylated reporter antibodies were then added robotically, thoroughly mixed, and incubated for an additional hour at room temperature. Multiplexes were developed using an excess of streptavidin-phycoerythrin solution which was thoroughly mixed into each multiplex and incubated for one hour at room temperature. The volume of each multiplexed reaction was reduced by vacuum filtration and the volume increased by dilution into matrix buffer for analysis. Analysis was performed in a Luminex instrument and the resulting data stream was interpreted using proprietary PlateReader data analysis software. Assays were run in high density multiplexed panels and the least detectable dose (LDD) was determined as the mean +3 standard deviations of 20 sample diluent readings. An eight (*n* = 8) point standard curve was used to obtain quantitative measurements for each sample. Standards and quality controls were run on each plate with a replicate of each standard positioned in the first and last column of the plate. Quality controls were run in duplicate along different points of the curve to ensure both accuracy and precision for each analyte. The analytes included in the assays and their LDD were: Brain-Derived Neurotrophic Factor (BDNF) [0.027 ng/mL], Eotaxin-1/CCL11 [67 pg/mL], Factor VII [3.3 ng/mL], Granulocyte-Macrophage Colony-Stimulating Factor (GM-CSF) [14 pg/mL], Intercellular Adhesion Molecule (ICAM)-1 3.3 [ng/mL], Interferon (IF)-γ [1.6 pg/mL], IL-1α [1.5 pg/mL], IL-1β [2.2 pg/mL], IL-1Ra [156 pg/mL], IL-2 [5.9 pg/mL], IL-3 [1.6 pg/mL], IL-4 [9.4 pg/mL], IL-5 [2.7 pg/mL], IL-6 [1.6 pg/mL], IL-7 [8.2 pg/mL], IL-8/CXCL8 [1.3 pg/mL], IL-10 [3.3 pg/mL], IL-12p40 [95 pg/mL], IL-12p70 [34 pg/mL], IL-15 [0.19 ng/mL], IL-17 [1.6 pg/mL], IL-18 [6.7 pg/mL], IL-23 [0.66 ng/mL], Macrophage Inflammatory Protein (MIP)-1α/CCL3 [18 pg/mL], MIP-1β/CCL4 [16 pg/mL], Matrix Metalloproteinase (MMP)-3 [26 pg/mL], MMP-9 [12 ng/mL], Monocyte Chemotactic Protein (MCP)-1/CCL2 [27 pg/mL], SCF [65 pg/mL], Tumor Necrosis Factor (TNF)-α [13 pg/mL], TNF-β [3.0 pg/mL], and VEGF [20 pg/mL].

Concentrations of leptin were determined using human leptin radioimmunoassay kits (Millipore) at the Metabolism Core at the Nutrition Obesity Research Center at the University of Alabama in Birmingham. The LDD of the leptin assays was 0.97 ng/mL and the inter-assay and intra-assay CVs were 4.18 % and 5.32 %, respectively.

### Data analyses

Survey data were collected from the Dooblo SurveyToGo studio software and merged with the main database independently by two investigators, and compared to ensure accuracy. All data analyses were performed using SPSS Statistics for Windows v21.0 (Armonk, NY: IBM Corp).

To examine serum inflammatory biomarker differences between GWI and HV, we tested group differences in the levels of cytokines over time. Group differences in cytokine concentrations were tested with generalized estimating equations (GEE). GEEs properly account for repeated measures within participants, and therefore allow for more precise estimates of individual- and group-means. GEEs were conducted in a univariate fashion. The participant identification number was used as a subject nesting variable, and the study day as the repeated measures index. Participant type (GWI or HV) was entered as the between-subjects factor.

Differences in between-group cytokine variability over the course of the immune monitoring period were also tested. Day-to-day cytokine fluctuations were used to calculate coefficients of variation (CVs) for each analyte, nested by individual. CV differences between the GWI and HV groups were tested with independent samples t-tests.

To test whether daily fatigue in GWI is related to fluctuations in cytokines, we used linear mixed models (LMM). LMMs can properly model effects with repeated measures, as can GEEs, but can also allow for intercepts and slopes to vary for each individual participant, by treating the participant as a random factor. Fatigue and cytokine data were person-centered (z-score-transformed) and temporally smoothed using a three-day moving average to improve pattern detection [50, 51]. Because only half of the HV individuals had measurable levels of fatigue, these analyses were performed on the GWI individuals only. For all applicable analyses, we determined significance using an α = 0.05 false-discovery rate to adjust for the 93 planned comparisons, yielding a *p =* 0.0098 threshold significance. For the cytokine that was most significantly associated with fatigue at the group-level, we also performed visual analyses of the relationship between each individual participant’s cytokine concentrations and fatigue symptoms over time.
